# Pre-existing disease: the most important factor for health related quality of life long-term after critical illness: a prospective, longitudinal, multicentre trial

**DOI:** 10.1186/cc8967

**Published:** 2010-04-15

**Authors:** Lotti Orwelius, Anders Nordlund, Peter Nordlund, Eva Simonsson, Carl Bäckman, Anders Samuelsson, Folke Sjöberg

**Affiliations:** 1 Departments of Intensive Care Linköping University Hospital, Medicine and Health Sciences, Faculty of Health Sciences, Linköping University, Garnisonsvägen, Linköping, 581 85, Sweden; 2 TFS Trial Form Support AB, Ruben Rausings gata 11B, Lund, 223 55, Sweden; 3 Department of Anaesthesia and Intensive Care, Ryhov Hospital, Jönköping, 551 85, Sweden; 4 Department of Anaesthesia and Intensive Care, Vrinnevi Hospital, Gamla Övägen 25, Norrköping, 601 82, Sweden; 5 Department of Intensive Care, Linköping University Hospital, Garnisonsvägen, Linköping, 581 85, Sweden; 6 Department of Intensive Care Linköping University Hospital, Clinical and Experimental Medicine, Faculty of Health Sciences, Linköping University, Garnisonsvägen, Linköping, 581 85, Sweden

## Abstract

**Introduction:**

The aim of the present multicenter study was to assess long term (36 months) health related quality of life in patients after critical illness, compare ICU survivors health related quality of life to that of the general population and examine the impact of pre-existing disease and factors related to ICU care on health related quality of life.

**Methods:**

Prospective, longitudinal, multicentre trial in three combined medical and surgical intensive care units of one university and two general hospitals in Sweden. By mailed questionnaires, health related quality of life was assessed at 6, 12, 24 and 36 months after the stay in ICU by EQ-5D and SF-36, and information of pre-existing disease was collected at the 6 months measure. ICU related factors were obtained from the local ICU database. Comorbidity and health related quality of life (EQ-5D; SF-36) was examined in the reference group. Among the 5306 patients admitted, 1663 were considered eligible (>24 hrs in the intensive care unit, and age ≥ 18 yrs, and alive 6 months after discharge). At the 6 month measure 980 (59%) patients answered the questionnaire. Of these 739 (75%) also answered at 12 month, 595 (61%) at 24 month, and 478 (47%) answered at the 36 month measure. As reference group, a random sample (n = 6093) of people from the uptake area of the hospitals were used in which concurrent disease was assessed and adjusted for.

**Results:**

Only small improvements were recorded in health related quality of life up to 36 months after ICU admission. The majority of the reduction in health related quality of life after care in the ICU was related to the health related quality of life effects of pre-existing diseases. No significant effect on the long-term health related quality of life by any of the ICU-related factors was discernible.

**Conclusions:**

A large proportion of the reduction in the health related quality of life after being in the ICU is attributable to pre-existing disease. The importance of the effect of pre-existing disease is further supported by the small, long term increment in the health related quality of life after treatment in the ICU. The reliability of the conclusions is supported by the size of the study populations and the long follow-up period.

## Introduction

There is increasing focus on Health-Related Quality of Life (HRQoL) after critical illness [1]. In a recent systematic review of relevant factors for the outcome of HRQoL, after care in an ICU, it was found that important predictors other than age and sex are severity of illness (Acute Physiology and Chronic health Evaluation (APACHE) score), admission (acute/elective), or length of stay (LoS) [2]. In the same review, it was also suggested that pre-existing impairment or disease may be important because they are known to affect HRQoL and therefore should be controlled for. It will not be possible to accurately estimate the HRQoL of ICU survivors, the impact on HRQoL among ICU survivors or to compare the HRQoL of ICU survivors with that of the general population unless pre-existing disease is accounted for [3-7]. Interestingly, few studies have adjusted for the effect of the pre-existing diseases.

We used a new technique, based on a control population adjusted for pre-existing diseases from the uptake area of the study hospitals in this prospective, multicentre study with 36 months of follow up. HRQoL was examined after care in the ICU to assess the importance of pre-existing disease. The effect on HRQoL has been examined further in conjunction with the factors previously thought to be important, such as age, sex, social factors, admission diagnosis, APACHE II score, LoS in ICU and in hospital, and time spent on a ventilator.

Given the nature of HRQoL instruments, we hypothesised (in line with our findings in our previous pilot study [5]) that pre-existing disease is the most important factor and that other factors related to intensive care such as APACHE II score, admission diagnosis, time on ventilator, and in ICU and duration of stay are of less importance.

In accordance with our pre-study hypothesis our main findings were: firstly, only a small improvement in HRQoL over time, up to 36 months post ICU was seen; secondly, ICU-related factors had little effect on the reported HRQoL; and, lastly, the overall most important factor for the decreased HRQoL reported by the patients in the long term was their pre-existing diseases.

## Materials and methods

### Design

This prospective, longitudinal multicentre study took place in three mixed medical-surgical ICUs in the southeast of Sweden: one university and two general hospitals. Patients with primary coronary disease, those recovering after heart surgery and neurosurgery, neonates or patients with burns are treated in other specialised units and were excluded. The ICUs each admit 500 to 750 patients annually. Nearly all the admissions to these three ICUs are emergencies and the most common primary diagnoses are multiple trauma, sepsis, and disturbances in respiratory or circulatory systems or both.

### Study population and reference group

All patients aged 18 years and older, who were admitted consecutively between 1 August 2000 and 30 June 2004, remained in the ICU for more than 24 hours, were alive six months after discharge from hospital and consented to participate in the study were included.

Patients who were readmitted were included only on their first admission. After the national Swedish Social Security register had been checked to avoid sending enquiries to patients who had died, we sent information and a request to participate to each patient by mail, together with a structured questionnaire and a preaddressed and prepaid return envelope. Patients who had not responded within 10 days were contacted by telephone by one of the investigators (LO, ES or CB). If the telephone contact or first mailing achieved no answer two reminders were sent out (at three and six weeks). The patients gave their informed consent prior to participating in the study.

Data from a public health survey of the county of Östergötland were used for comparison of HRQoL and pre-existing disease. This reference group consisted of a random sample of the general population living in the uptake area of the hospitals. That survey was approached for the purpose of monitoring the general health of the reference group population in a different study and was completed during 1999 [8]. Questionnaires were initially sent out to 10,000 people aged 20 to 74 years. After two reminders, 6093 (61%) had responded [8].

The clinical databases in each hospital were used to extract data on age, sex, admission diagnosis, APACHE II score, LoS in ICU and hospital, time spent on the ventilator and outcome. The patients were categorised into diagnostic categories according to the main reason for admission: multiple trauma, sepsis, gastrointestinal, respiratory and other.

The study was approved by the Committee for Ethical Research at the University of Health in Linköping.

### Questionnaires and instruments

A set of structured questionnaires were mailed to the study population at 6, 12, 24 and 36 months after discharge from hospital. The questionnaire contained questions about the patients' background (employment, listed sick or not, born in Sweden or not, and pre-existing disease self-reported diagnosis). The questionnaire also asked, 'Have you had any significant illness, reduced body function or other medical problem and have had it for more than six months prior to the ICU period?' with the answer options of 'yes' or 'no'. Further, this question also had the pre-specified illnesses alternatives: 'cancer, diabetes, heart failure, asthma/allergy, rheumatic-gastrointestinal, blood, kidney, psychiatric, neurological disease, thyroid or any other metabolic disturbance, or any other long-term illness'. The last alternative was an open question with a slot for free text.

The instruments chosen for the evaluation of HRQoL were EuroQol 5-Dimensions (EQ-5D) questionnaire [9,10] and medical outcome Short Form health survey (SF-36) [11,12]. Both are known internationally and have been recommended for measuring HRQoL in critical care [1] although EQ-5D has not been validated in the ICU population. The EQ-5D is developed and applied by an international multidisciplinary research group from seven Scandinavian countries. The instrument is therefore validated in a Swedish population [13]. The EQ-5D involves a health state classification scheme of five items (mobility, self-care, usual activities, pain/discomfort and anxiety/depression), each having three alternatives (1 = no problems, 2 = moderate problems, and 3 = severe problems). The combination of answers on the five items represents the health state, ranging from 0 (worst possible health state) to 1.0 (best possible health).

SF-36 has reliability and validity in the ICU population [12,14]. It has been translated into Swedish and validated in a representative sample of the population [11,15]. It has 36 questions and generates a health profile of eight sub-scale scores: physical functioning, role limitations due to physical problems, bodily pain, general health, vitality, social functioning, role limitations due to emotional problems and mental health [15]. The scores on all sub-scales are transformed to a scale from 0 (the worst score) to 100 (best score) [16].

The questionnaire to the reference group also included questions on background characteristics as above, and questions about HRQoL ((EQ-5D and Medical outcome Short-Form health survey (SF-36)questionnaire) and health problems. Details and the method for this part has been previously discussed [5] .

### Statistical analysis

Data are presented as mean, median and 95% confidence intervals (CI). Unadjusted two-sample comparisons (Pearson's chi squared and Student's t test) were used to assess differences in background characteristics between the groups as appropriate. In the comparison of HRQoL (EQ-5D and SF-36) between the reference group and the study group at different occasions (6, 12, 24 and 36 months) a t-test (mean) and Wilcoxon (median) was used. A general linear model (GLM) was used to analyse the impact of background and ICU-related factors on HRQoL. Marginal means were estimated from the model including all statistically significant (*P *< 0.050) variables. To maximise the statistical power, the six-month follow-up data was used for this purpose (n = 980). Partial F were used to assess differences in diagnoses groups regarding HRQoL. GLM was also used to assess changes in HRQoL over time within groups. In analyses, comparing HRQoL over time, only survivors with answers at the follow up involved in the comparison were used (n = 478). Further, when ICU survivors were compared with the reference group, survivors older than 75 years were excluded because the reference population did not include subjects older than 75 years. This comparison was performed on the six-months data (n = 780) in relation to the follow-up data with the responders in all four occasions (n = 388). No adjustments for multiple testing were performed in this study and *P *values were regarded as descriptive. Findings were considered significant; however, only if there were concurrent changes in several related variables. A *P *value lower than 0.05, were considered as an indication of a statistically important finding.

The Statistical Package for the Social Sciences (version 15.0; SPSS Inc., Chicago, IL, USA) was used for the statistical analyses.

## Results

### Study population

**Figure 1:**
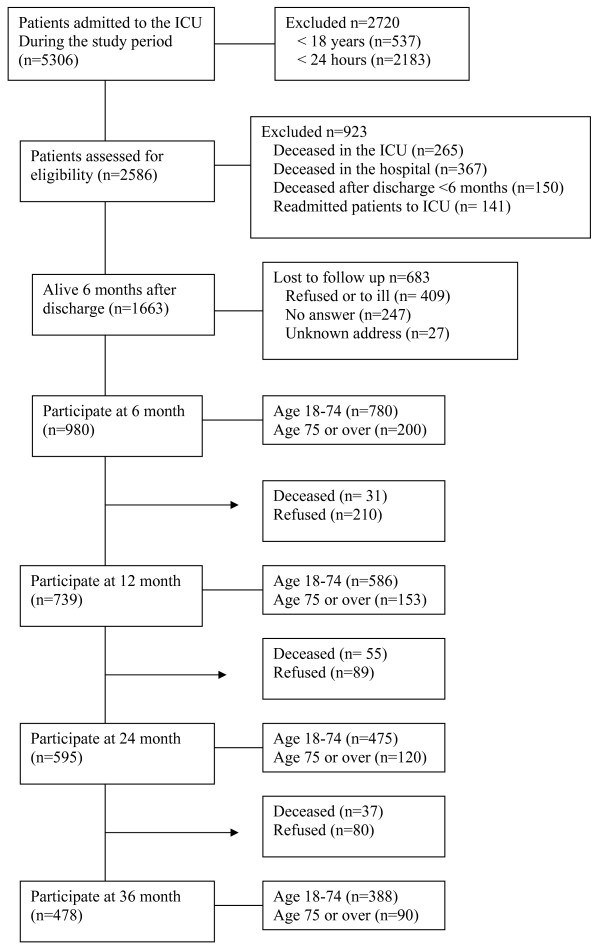
**Outline of the study protocol**.

A total of 1,663 patients met the inclusion criteria. After two remainders, 980 patients (59%) answered the questionnaire at six months. Of these 739 (75%) also answered at 12 months, 595 (61%) at 24 months and 478 (47%) at 36 months (Figure 1). During the study period 123 (12%) patients died and 379 (39%) patients were lost to follow up (Figure 1).

The group who did not respond at all in the study (n = 683) differed from the group who responded in that there were fewer men (*P *= 0.02), higher average APACHE II score (*P *= 0.04), shorter LoS in the ICU (*P *< 0.0001), shorter time on ventilator (*P *< 0.0001), and fewer gastrointestinal admission diagnoses (*P *= 0.02; Table 1).

**Table 1:** Clinical details

	Answered on all four occasions	Withdrawals between 6 and 36 months		Answered on 6 months	Non-responders		Alive 6 months after discharge
**Variable**	**(n = 478)**	**(n = 502)**	***P *a**	**(n = 980)**	**(n = 683)**	***P *b**	**(n = 1663)**
							
Male/female	274/204 (57)	292/210 (58)	0.32	567/413 (58)	357/326 (52)	0.02	924/739 (55.6)
Age (years)	58.8 (17.0)	57.6 (19.3)	0.30	58.2 (18.2)	57.7 (19.6)	0.54	58.0 (18.8)
APACHE II score	15.3 (7.2)	15.9 (8.1)	0.22	15.6 (7.7)	16.3 (7.6)	0.04	15.9 (7.6)
Stay in ICU (hours)	126.6 (173.9)	119.7 (161.9)	0.52	123.1 (167.8)	93.1 (105.5)	<0.001	110.9 (146.3)
Stay in hospital (days)	15.5 (20.1)	14.6 (19.2)	0.46	15.0 (19.6)	14.8 (19.9)	0.85	14.9 (19.9)
Time on ventilator (hours	68.1 (165.5)	56.2 (142.4)	0.23	62.0 (154.2)	33.5 (81.6)	<0.001	50.2 (130.0)
Diagnosis on admission to ICU			0.80			0.02	
Multiple trauma	57 (12)	57 (11)		117 (12)	69 (10)		186 (11)
Sepsis	37 (8)	47 (9)		82 (8)	53 (8)		135 (8)
							
Gastrointestinal disease	101 (21)	100 (20)		204 (21)	104 (15)		308 (18)
Respiratory disease	85 (18)	112 (22)		196 (20)	147 (22)		343 (21)
Miscellaneous	198 (41)	186 (37)		381 (39)	309 (45)		690 (42)
Pre-existing diseases				725 (74)			
Cancer				116 (16)			
Diabetes				131 (13)			
Cardiovascular				203 (28)			
							
Gastrointestinal				122 (17)			
Miscellaneous				628 (64)			
Number of diseases							
0				256 (26.1)			
1				418 (42.7)			
2				190 (19.4)			
≥ 3				116 (11.8)			

a, between groups answered at all occasions and withdrawals between 6 and 36 months; b, between groups answered at 6 months and non-responders

APACHE II: Acute Physiology and Chronic health Evaluation.

Data are number (%) or mean (standard deviation)

The clinical characteristics of patients in the final study population (e.g., the patients who answered at the 6-, 12-, 24- and 36-months follow ups) and the patients who participated at some time but did not complete the whole study is shown in Table 1. There were no significant differences in sex, age, APACHE II score, LoS in the ICU and in hospital, time treated on a ventilator, or diagnosis at admission among the two groups of patients. For the patients who answered at six months, 724 (74%) had pre-existing disease.

For the reference group, questionnaires were initially sent out to 10,000 people. After two reminders, 6,093 (61%) had responded [8]. Apart from lower percentages of immigrants and single households, the responders in the reference group differed only marginally from the reference population of the county [8]. The reference group were younger (*P *< 0.001), had a higher rate of women (*P *< 0.001), higher rate of employment (*P *< 0.001), and had a lower rate of comorbidity (51%; *P *< 0.001) than the ICU group (n = 980; data not shown).

### Determinants of HRQoL

The general linear model was used to evaluate the effect of baseline variables (age, sex, sick leave before ICU, marital status, employment before ICU, employment at the follow-up time, education, born in Sweden, and pre-existing disease) and ICU-related factors on HRQoL based on the six months measure (APACHE II, LoS ICU, LoS hospital, diagnosis on admission to ICU, time on ventilator). In these analyses APACHE II score and duration of stay in ICU, and time on ventilator showed no association with HRQoL, whereas pre-existing disease, diagnosis at admission (trauma), duration of stay in hospital, born in Sweden, sick leave before ICU, employment before ICU (not employed), sex (female)and age did [see Additional file 1].

### Health-related quality of life over time

#### EQ-5D

Mean and median EQ-5D scores for the reference group and ICU survivors (<75 years) who answered the questionnaire at all four occasions (n = 388) are shown in Table 2. There were statistically significant differences between them, and the difference was in the range of 0.15 to 0.18. No increases over time in the EQ-5D values were seen for the ICU survivors.

**Table 2:** Health-related quality of life (EQ-5D) ICU patients aged younger than 75 years, answered at 6, 12, 24, and 36 months after discharge (n = 388) and reference group (n = 6093) data

		ICU group (months)				
			
	Reference group	6	12	24	36	*P *value
EQ-5D						
Number	6093	388	388	388	388	
Mean	0.84	0.66	0.68	0.68	0.69	<0.001 †
95% CI	0.83 to 0.84	0.63 to 0.69	0.65 to 0.71	0.65 to 0.71	0.65 to 0.72	
Median	0.85	0.72	0.73	0.73	0.73	<0.001 ‡
Pre-existing disease						
Number (%)	3095 (51)	268 (69)	268 (69)	268 (69)	268 (69)	
Mean	0.75	0.59	0.62	0.61	0.62	<0.001†
95% CI	0.7 to 0.76	0.55 to 0.63	0.58 to 0.65	0.57 to 0.65	0.58 to 0.66	
Median	0.80	0.69	0.72	0.72	0.72	<0.001 ‡
Healthy						
Number (%)	2998 (49)	120 (31)	120 (31)	120 (31)	120 (31)	
Mean	0.92	0.81	0.83	0.84	0.82	<0.001 †
95% CI	0.92 to 0.93	0.77 to 0.85	0.79 to 0.87	0.80 to 0.88	0.79 to 0.86	
Median	1.0	0.80	0.85	0.85	0.82	<0.001 ‡

Study group compared with reference group; † *P *value for mean (T-Test), ‡ *P *value for median (Wilcoxon).

CI: confidence interval; EQ-5D: EuroQol 5-Dimensions questionnaire.

The significant differences remained, although smaller, when comparisons were made between those in both groups (ICU and reference) that either had pre-existing diseases or had been previously healthy (Table 2). The overall mean difference in EQ-5D was 0.16 with and without pre-existing disease at all four occasions, in the group with pre-existing disease it was 0.14 and in the previously healthy group it was 0.10. Regarding comparison in the ICU patients only the difference in EQ-5D was 0.21 between the group with pre-existing disease and the previously healthy group. Patients in the previously healthy group had higher scores at all times than the patients with pre-existing diseases (*P *< 0.0001). Again no increases in EQ-5D were seen over time for these two groups.

#### SF-36

For the ICU survivors (n = 388) improvement over time was minor (Table 3). There were no statistically significant changes in any SF-36 dimensions mean-score apart from physical function between 6 and 24 months with improvements from 66.3 to 70.1 (*P *= 0.002), physical role functioning between 6 and 12 month with improvements from 47.8 to 56.5 (*P *< 0.001), and social function between 6 and 12 months with improvements from 73.0 to 76.9 (*P *= 0.008).

**Table 3:** Health-related quality of life for the ICU patients aged younger than 75 years, answered at 6, 12, 24 and 36 months after discharge (n = 388) and reference group (n = 6093)

			ICU patients	ICU patients	ICU patients	ICU patients	
				
		Reference group	6 months	12 months	24 months	36 months	*P *value
SF-36		(n = 6093)	(n = 388)	(n = 388)	(n = 388)	(n = 388)	
PF	Mean	87.86	66.29	68.20	70.07	68.76	<0.001 †
	SD	19.29	29.15	29.53	28.41	28.90	
	CI (95%)	87.38:88.35	63.35:69.22	65.24:71.16	67.23:72.91	65.88:71.65	
	Median	95.0	75.0	75.0	75.0	75.0	<0.001 ‡
RP	Mean	82.63	47.78	56.51	57.77	59.21	<0.001 †
	SD	33.06	44.48	43.95	42.92	43.07	
	CI (95%)	81.79:83.48	43.29:52.27	52.08:60.95	53.46:62.07	54.87:63.55	
	Median	100	50.0	75.0	75.0	75.0	<0.001 ‡
BP	Mean	73.70	62.34	63.93	64.38	63.98	<0.001 †
	SD	25.47	29.68	29.12	29.15	30.07	
	CI (95%)	73.06:74.34	59.35:65.33	61.01:66.86	61.47:67.30	60.98:66.98	
	Median	84.0	62.0	62.0	62.0	62.0	<0.001 ‡
GH	Mean	73.10	57.75	59.83	58.38	58.41	<0.001 †
	SD	21.52	24.01	25.17	25.91	25.60	
	CI (95%)	72.55:73.65	55.33:60.18	57.30:62.36	55.78:60.98	55.86:60.97	
	Median	77.0	57.0	62.0	57.0	57.0	<0.001 ‡
VT	Mean	65.75	56.18	58.43	57.08	56.64	<0.001 †
	SD	22.52	24.62	23.94	23.96	24.46	
	CI (95%)	65.18:66.32	53.70:58.65	56.03:60.84	54.68:59.48	54.20:59.08	
	Median	70.0	55.0	60.0	60.0	55.0	<0.001 ‡
SF	Mean	86.68	73.00	76.92	76.62	75.39	<0.001 †
	SD	21.03	26.94	25.36	26.12	26.04	
	CI (95%)	86.15:87.21	70.29:75.71	74.38:79.47	74.00:79.23	72.79:77.99	
	Median	100	75.0	87.5	87.5	81.2	<0.001 ‡
RE	Mean	85.36	68.01	69.76	69.38	71.71	<0.001 †
	SD	30.25	41.55	40.74	40.98	39.99	
	CI (95%)	84.58:86.13	63.77:72.25	65.64:73.89	65.25:73.51	67.68:75.74	
	Median	100	100	100	100	100	<0.001 ‡
MH	Mean	78.82	71.88	73.79	72.88	72.19	<0.001 †
	SD	18.69	21.93	20.59	21.76	21.09	
	CI (95%)	78.35:79.30	69.67:74.08	71.72:75.85	70.70:75.06	70.08:74.29	
	Median	84.0	76.0	80.0	80.0	76.0	<0.001 ‡

Study group compared with reference group; † *P *value for mean (T-Test), ‡ *P *value for median (Wilcoxon)

BP, bodily pain; CI, confidence interval; GH, general health; MH, mental health; PF, physical functioning; RE, role limitations due to emotional problems; RP, role limitations due to physical problems; SD, standard deviation; SF, social functioning; SF-36, short form health outcome;VT, vitality.

The reference group scored significantly higher HRQoL than the study group in all dimensions of the SF-36 (*P *< 0.001), with mean score differences between 6.9 (mental health) to 34.8 (physical role functioning) at the six-month measure.

**Figure 2:**
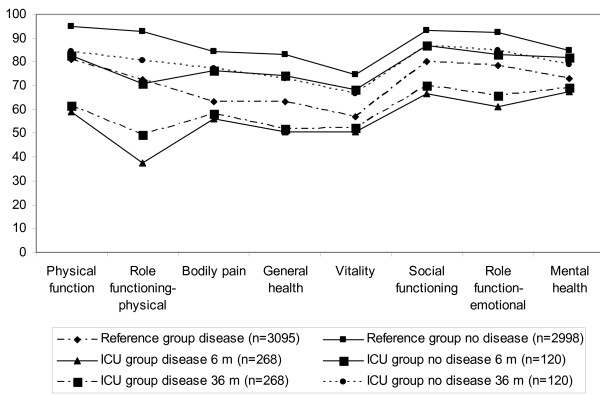
**Medical Outcome Short Form results**. Results from the eight scales in the reference group with diseases (n = 3095) and the healthy group (n = 2998), compared with the ICU group aged 18 to 74 years at the 6 and 36 month measures, either with diseases (n = 268) or with no disease (n = 120) who answered at all occasions (n = 388).

In Figure 2, the study group and reference group are divided into the previously healthy and those having pre-existing diseases (the measure at 6 and 36 months are shown). The patients who were healthy before the ICU period (n = 120) and the healthy reference group (n = 2998) were significantly different (*P *< 0.005) in all eight dimensions at all times apart from mental health at six months (*P *= 0.2). The mean differences in SF-36 scale scores were in the range between 6.1 (mental health) to 27.3 (role physical), in the group with pre-existing disease it was from 3.7 (mental health) to 27.5 (role physical) and in the previously healthy group it was from 4.3 (mental health) to 15.1 (role physical).

When the ICU patients with pre-existing disease (n = 268) were compared with the reference group who had diseases (n = 3,095) statistically significant differences (*P *< 0.04) were seen in all eight dimensions over time apart from mental health at 12 months (*P *= 0.07; not shown in Figure). Figure 2 also shows that those in the reference group with diseases had reduced HRQoL in six of eight dimensions in SF-36 (not physical functioning and role physical) compared with the study group who were healthy before the intensive care period.

#### HRQoL among patients dying during the follow up

In total, 139 patients who were included in the study died during the follow up. They answered the HRQoL enquiry at 6 and 12 months, or at 24 months after discharge from the hospital. These patients, with the highest frequency of pre-existing diseases, had the lowest HRQoL scores registered in the study (data not shown).

## Discussion

Data from this study shows the large impact of pre-existing disease on HRQoL and the importance of accounting for pre-existing disease when the HRQoL of ICU survivors is studied. Four important and novel observations were noted in this study:

First, pre-existing disease seems to be the most important factor overall for long-term HRQoL after a critical illness and a period of critical care. In this study the only factor that affected all dimensions in the HRQoL outcome was pre-existing disease (EQ-5D and all eight dimensions in SF-36). Furthermore, the size of this effect was most often in the range of 15 to 20 scale units (SF-36). This is to be compared with the other factors examined where such large effects were not at all registered. It is important to stress that a clinically significant effect is claimed for a change larger than five scale units [17]. To our knowledge, this is the first time the effect of pre-existing disease has been addressed in a systematic way in ICU-related outcome research. Although claimed to be an important factor in other studies, it was then not specifically examined and adjusted for [2,18,19]. When we exclude the factor 'pre-existing diseases' from the analyses, an increasing number of significant results were found for the ICU-related variables as has been presented by others [3,20-23] (data not shown).

Secondly, there were only few and minor improvements over time in HRQoL assessed by EQ-5D and SF-36. Data from SF-36, showed only clinically significant (>5%) [17] improvements in role limitations due to physical problems. In our study we found no effects on HRQoL by ICU-related factors. This finding supports the lack of long-term improvement related to the specifics of the critical care event. Furthermore, the minor improvement, albeit not clinically relevant, that was noted continued up to two years after the period in the ICU, which is longer than the six months claimed by others [1], but in line with Cuthbertson and colleagues in their five-year follow-up study [24]. It needs then to be stressed that, Dowdy and colleagues [2], in 2005, pointed out that median follow-up time after critical illness in the studies they reviewed was only six months. Since then, we have found only one study in general ICU patients with a longer follow-up period after critical illness than 12 months [24]. Several investigations examining HRQoL changes over time for intensive care patients include all patients responding at each occasion. This introduces a possible error in that it may falsely improve HRQoL outcome over time. Such an improvement is due to the loss of those dying early during the follow-up period (being more ill, having a higher rate of pre-existing diseases and with a lower HRQoL) leaving the patients with a better HRQoL. This effect was also found in our data although the subgroups were small (Figure 1).

Thirdly, and in line with our pre-study hypothesis, our data support that the effects of ICU-related factors (APACHE II score, admission diagnosis, time on ventilator, duration of stay in ICU and hospital) are minor. Results from other studies, however, indicate that various ICU-related factors affect HRQoL after intensive care measured by EQ-5D or SF-36. Kleinpell [20] and Vedio and colleagues [25] found significant associations between APACHE II score and poor physical function or general health. For admission diagnosis, few studies report differences between medical and surgical diagnosis but patients who survive trauma injuries had significantly worse pain or discomfort ratings on EQ-5D than did other survivors after ICU care up to 18 months after discharge [3,4,21]. We also recorded significant influence by trauma on bodily pain. Previous studies have found that time spent on the ventilator [26] or LoS in the ICU [22,26] or the hospital [7] reduced HRQoL by up to 12 months after critical illness. None of the studies cited above included pre-existing diseases in their analysis.

Lastly, it may be stressed that this study based on HRQoL data gathered by two different, separate and validated HRQoL instruments, EQ-5D and SF-36, show similar HRQoL outcome profiles. Furthermore, the study contains a considerable number of ICU patients in the study group (n = 980) and has in addition a relatively long follow-up time (36 months). In these three aspects it is probably the most sizeable study for this group of general ICU patients seen yet, which supports the value of the findings.

When we aim to adjust for pre-existing diseases it is important to find relevant control groups. Most often those adjusted for age and sex are used [22,23,25]. However, they may not be adequate from the perspective of pre-existing disease, because the prevalence of pre-existing disease is significantly higher among patients in ICU [3,5,7,23], and pre-existing disease reduces HRQoL [3-5,7,27]. Using healthy reference groups adjusted only for age and sex then leads to a faulty interpretation of the HRQoL values among former ICU patients as their HRQoL may be assumed to be lower prior to the admittance to the ICU due to their pre-existing diseases. Such comparisons are, however, seen in most studies [7,18,22-25]. The present study was therefore constructed so that we used a large reference population selected from the uptake area of the hospitals and particularly adjusted for comorbidity. This was practically feasible as the Division of Preventive and Social Medicine and Public Health Science in parallel made a general health survey in the county (1 million inhabitants), which assessed comorbidity and their effects on HRQoL in a large group encompassing 10,000 people [8].

The present study does not take into account the seriousness of pre-existing diseases and the burden of each disease [28]. We think that part of the differences that remained after the adjustment for pre-existing diseases is the result of such an effect. This needs to be addressed more thoroughly in future studies. One interesting finding is that the previously healthy persons that were cared for in an ICU ended up with a HRQoL after ICU stay that is almost identical to the group in the reference population that has comorbidity. Assuming that the event at the ICU has lead to the patient obtaining a disease or impairment that has a chronic profile almost all of the ICU-related HRQoL decrease for this group may thus be explained. Therefore, special interest for future HRQoL investigations needs to focus on the specific diagnoses or effects that affect the patient during the ICU treatment period.

## Conclusions

This study, based on the comparison of HRQoL data obtained from a sizeable, multicentre, long-term follow up of ICU survivors and a large cohort of inhabitants living in the uptake areas of the hospitals, confirms that pre-existing disease have a larger impact on HRQoL than ICU or psychosocial factors. Furthermore, the data show that ICU survivors do not experience any significant increase in their HRQoL after six months and only minor improvement are registered up to 36 months after discharge from ICU and hospital. These findings underline the importance of accounting for pre-existing diseases when HRQoL is studied in former ICU patients.

## Key messages

• The most important factor for the low HRQoL status reported long term by former ICU patients was their pre-existing diseases

• ICU-related factors had little effect on the reported HRQoL

• Only minor improvements in HRQoL over time, up to 36 months post ICU was seen

## Abbreviations

APACHE II: Acute Physiology and Chronic health Evaluation score; EQ-5D: EuroQol 5-Dimensions; HRQoL: Health-Related Quality of Life; LoS: length of stay; SF-36: Short Form health survey.

## Competing interests

The authors declare that they have no competing interests.

## Authors' contributions

LO designed the study, performed and interpreted the data analysis, and drafted the manuscript. AN and FS designed the study and interpreted the data analysis. ES and CB collected the data and revised the manuscript. PN and AS revised the manuscript. All authors have read and approved the final manuscript.

## Supplementary Material

Additional file 1 **Multivariate regression analysis (general linear model (GLM)) mean score**. Word file containing multivariate regression analysis (GLM) mean score with significant variables from the univariate analysis and Health-Related Quality of Life (HRQoL) at six months (n = 980). Click here for file
