# Supplementary material for: Pre-existing disease: the most important factor for health related quality of life long-term after critical illness: a prospective, longitudinal, multicentre trial
Source: Crit Care. 2010 Apr 15;14(2):R67. doi: 10.1186/cc8967 (PMC2887189; doi:10.1186/cc8967)
Supplement: Additional file 1 — Multivariate regression analysis (general linear model (GLM)) mean score. Word file containing multivariate regression analysis (GLM) mean score with significant variables from the univariate analysis and Health-Related Quality of Life (HRQoL) at six months (n = 980). [file cc8967-S1.DOC]

**Additional file 1**

|  | EQ-5D |  | Bodily |  | General |  | Social |  | Role |  | Mental |  | Physical |  | Role |  | Vitality |
| --- | --- | --- | --- | --- | --- | --- | --- | --- | --- | --- | --- | --- | --- | --- | --- | --- | --- |
| Variable |  |  | pain |  | health |  | function |  | emotional |  | health |  | function |  | physical |  |  |
|  |  |  |  |  |  |  |  |  |  |  |  |  |  |  |  |  |  |
| Preexisting disease |  | <.001 |  | <.001 |  | <.001 |  | <.001 |  | <.001 |  | <.001 |  | <.001 |  | <.001 |  |
| Yes | 0.56 |  | 55.7 |  | 47.3 |  | 62.2 |  | 48.9 |  | 64.0 |  | 56.9 |  | 43.0 |  | 52.4 |
|  | (0.52:0.60) |  | (52.4:59.1) |  | (44.5:50.1) |  | (57.8:66.7) |  | (41.4:56.4) |  | (60.3:67.6) |  | (54.6:59.1) |  | (39.5:46.5) |  | (50.4:54.4) |
| No | 0.76 |  | 72.2 |  | 68.1 |  | 76.7 |  | 63.7 |  | 72.2 |  | 77.4 |  | 69.1 |  | 67.4 |
|  | (0.70:0.81) |  | (67.8:76.5) |  | (64.0:72.2) |  | (71.1:82.4) |  | (54.3:73.1) |  | (67.6:76.8) |  | (73.8:80.9) |  | (63.5:74.6) |  | (64.2:70.6) |
| Diagnosis |  | 0.002 |  | <.001 |  | NS |  | NS |  | NS |  | NS |  | NS |  | NS |  |
| Multiple trauma | 0.60 |  | 55.6 |  |  |  |  |  |  |  |  |  |  |  |  |  |  |
|  | (0.52 0.67) |  | (49.1:62.1) |  |  |  |  |  |  |  |  |  |  |  |  |  |  |
| Sepsis | 0.59 |  | 61.0 |  |  |  |  |  |  |  |  |  |  |  |  |  |  |
|  | (0.52:0.67) |  | (53.8:68.1) |  |  |  |  |  |  |  |  |  |  |  |  |  |  |
| Gastrointestinal | 0.73 |  | 66.3 |  |  |  |  |  |  |  |  |  |  |  |  |  |  |
|  | (0.67:0.78) |  | (61.5:71.2) |  |  |  |  |  |  |  |  |  |  |  |  |  |  |
| Respiratory | 0.68 |  | 66.4 |  |  |  |  |  |  |  |  |  |  |  |  |  |  |
|  | (0.62:0.73) |  | (61.4:71.5) |  |  |  |  |  |  |  |  |  |  |  |  |  |  |
| Miscellaneous | 0.70 |  | 70.5 |  |  |  |  |  |  |  |  |  |  |  |  |  |  |
|  | (0.65:0.74) |  | (66.5:74.4) |  |  |  |  |  |  |  |  |  |  |  |  |  |  |
| LoS hospital |  | NS |  | NS |  | NS |  | NS |  | NS |  | NS |  | NS |  | 0.03 |  |
| <5 days |  |  |  |  |  |  |  |  |  |  |  |  |  |  | 59.8 |  |  |
|  |  |  |  |  |  |  |  |  |  |  |  |  |  |  | (54.3:65.2) |  |  |
| 6-13 days |  |  |  |  |  |  |  |  |  |  |  |  |  |  | 58.1 |  |  |
|  |  |  |  |  |  |  |  |  |  |  |  |  |  |  | (53.0:63.2) |  |  |
| >13 days |  |  |  |  |  |  |  |  |  |  |  |  |  |  | 50.3 |  |  |
|  |  |  |  |  |  |  |  |  |  |  |  |  |  |  | (44.9:55.7) |  |  |
| Born i Sweden |  | NS |  | NS |  | NS |  | 0.004 |  | <.001 |  | 0.001 |  | NS |  | NS |  |
| Yes |  |  |  |  |  |  | 74.7 |  | 67.1 |  | 72.8 |  |  |  |  |  |  |
|  |  |  |  |  |  |  | (71.2:78.1) |  | (61.2:72.9) |  | 70.0:75.6) |  |  |  |  |  |  |
| No |  |  |  |  |  |  | 64.3 |  | 45.6 |  | 63.3 |  |  |  |  |  |  |
|  |  |  |  |  |  |  | (56.8:71.8) |  | (33.2:57.9) |  | (57.3:69.4) |  |  |  |  |  |  |
| Sick leave before ICU |  | 0.04 |  | NS |  | 0.01 |  | 0.01 |  | 0.01 |  | 0.01 |  | NS |  | NS |  |
| Yes | 0.62 |  |  |  | 53.9 |  | 65.1 |  | 50.0 |  | 64.8 |  |  |  |  |  |  |
|  | (0.65:0.69) |  |  |  | (48.4:59.3) |  | (58.0:72.2) |  | (38.4:61.3) |  | (59.1:70.5) |  |  |  |  |  |  |
| No | 0.69 |  |  |  | 61.5 |  | 73.9 |  | 62.8 |  | 71.4 |  |  |  |  |  |  |
|  | (0.69:0.72) |  |  |  | (59.7:63.4) |  | (70.2:77.6) |  | (56.4:69.1) |  | (68.4:74.3 |  |  |  |  |  |  |
| Employment before ICU |  | NS |  | 0.01 |  | NS |  | NS |  | NS |  | NS |  | NS |  | NS |  |
| Employed/leader |  |  | 68.7 |  |  |  |  |  |  |  |  |  |  |  |  |  |  |
|  |  |  | (64.8:72.6) |  |  |  |  |  |  |  |  |  |  |  |  |  |  |
| Unemployed |  |  | 62.5 |  |  |  |  |  |  |  |  |  |  |  |  |  |  |
|  |  |  | (54.9:70.2) |  |  |  |  |  |  |  |  |  |  |  |  |  |  |
| Retired |  |  | 60.6 |  |  |  |  |  |  |  |  |  |  |  |  |  |  |
|  |  |  | (56.8:64.4) |  |  |  |  |  |  |  |  |  |  |  |  |  |  |

| Adjusted for age and sex; NS, Non significant |  |  |  |  |  |  |  |  |
| --- | --- | --- | --- | --- | --- | --- | --- | --- |
| HRQoL, health related quality of life; EQ-5D, EuroQoL 5 Dimensions questionnaire;  CI, Confidence interval; LoS, length of stay | | | | | | | | |
